# Nervous Diseases of Women

**Published:** 1873-10

**Authors:** 


### Nervous Diseases of Women.

In an article on Nervous Diseases of Women (*Archives of Scientific and Practical Medicine*), Dr. Wm. B. Neftel, says: In concluding this article on nervous diseases of women, I wish to allude to some ætiological and therapeutical peculiarities as regards diseases of women generally. Much has been said already with reference to the irrational mode of woman's clothing impeding the functions of the organs of the thoracic and abdominal cavities, and predisposing to different diseases. It is to be regretted, however, that this subject should have been discussed mostly by social reformers, moralists, or by advocates of a special cure of female diseases. The only competent judges in this matter are the physicians, and the most decisive arguments for the discussion must be derived from *post-mortem* examinations and from experiments. In this respect we possess some facts that may serve as a basis for further investigation. I have seen at the autopsies of women in Virchow's Pathological Institute some specimens of livers presenting remarkable deformities in consequence of tight lacing. Such a liver bears the name of corset-liver (Schneurleber), and illustrations of such deformities can be seen in Frerich's classical work on diseases of the liver.† This malformation must necessarily interfere with the normal functions of this important organ, and must be followed by chronic and incurable derangements of the digestion and of the circulation in the system of the portal vein, with all their consequences.

In 1863 I made a considerable number of experiments on animals in the laboratory of Prof. Harley, in the University College, London, which I continued, in 1867, in the Pathological Institute

†Frerich's Klinik der Leberkrankheiten Zweite Aufl. Bd. I., figs. 7, 8, 9, 10, 11.

of Prof. Virchow. Though the object of these experiments was to study the effect of a slight but continuous impediment of the respiratory movements upon the development of affections of the lungs and heart, I found that the effect of the pressure was still more pronounced in the abdominal organs, especially the liver and kidneys. I have not yet published the result of these experiments, and give here the following extract from my note-book:

Rabbits cannot bear even a very slight compression of the trunk by a bandage. Their respiration becomes accelerated, the conjunctiva congested, and they invariably die, sometimes after twenty-four hours. May 22d, 1867, I applied loosely a bandage around the thorax and abdomen of a rabbit. The animal died May 26th. I found the liver and kidneys very much congested. In the liver, especially on its convex surface, numerous white spots, from the size of a pin's head to that of a pea, very resistant and retracted. There were, besides, psorospermia.

May 29th, 1867, a similar bandage, but still looser than the former, was applied to a well-nourished and vigorous rabbit. which, nevertheless, died June 11th, being much emaciated, and the liver and kidneys very much congested.

May 30th, 1867, I applied a very loose bandage around the thorax and partly around the abdomen of a female dog. During the first fortnight no perceptible change could be noticed in the animal; afterward it grew thinner, and coughed, which I ascribed to the irritating influence of the air of the chemical laboratory where the dog was kept. It died July 4th. At the *post-mortem* the blood was found very thin and dark, the heart dilated, the lungs emphysematous, a hæmorrhagic infarction in the spleen, and the small intestines contracted, the liver and kidneys congested, the epithelium of the latter fatty, degenerated, etc. In cases where the compression by the bandages was minimal, and the animal survived a longer time, the kidneys presented the appearance of Bright's disease in its first stages.

Perhaps it is worth mentioning that high-heeled shoes are very injurious, by excluding certain groups of muscles from participating in walking, and over-exerting other sets. Backache in the lumbar region is apt to follow after wearing such shoes for a considerable length of time.

That irregularities in the sexual functions play an important part in the ætiology of diseases of women is well known to physicians. I was told that not unfrequently ice-water is injected into the vagina immediately after sexual intercourse, with the object of preventing conception. Two ladies who were in the habit of so doing are suffering from large fibroid tumors of the uterus; others are affected with chronic metritis and extreme nervousness.

The injurious effect of the intense and sudden cold upon the sexual organs during their increased activity may easily account for their disorders. Moreover, admitting the development of tumors from local irritative processes, we can also explain the origin of the fibro-myomata in the above cases.

In treating chronic diseases of women, especially nervous disorders, we have to bear in mind the important influence of muscular exercise upon the healthy condition of the whole economy. The accumulated products of tissue-metamorphosis act in a deleterious manner upon all the vital processes, especially producing a depressing effect upon the muscular and nervous system, and a feeling of exhaustion. Ranke* has shown that these so-called exhausting substances (ermudende Stoffe,) as lactic acid, carbolic acid, etc., abolish the irritability and electro-motor properties of the muscles. Their effect can be neutralized by injecting blood through the blood vessels into the exhausted muscles. In the living organism it is the alkaline blood and lymph that act in the same beneficial way. The circulation of blood and lymph facilitate the removal from the muscles, and nervous apparatus, of these effete substances, and their final elimination from the system, after which these organs regain their irritability and other electro-motor properties. Thus we see persons tired and prostrated without any muscular activity, feel refreshed and vigorous in walking, and the more so, the more they continue to take exercise.

Furthermore, the neglect of exercising and developing the muscles, which constitute the largest part of the body, is followed by other injurious consequences for the whole organism. During muscular activity a large amount of blood circulates in the muscles, thus relieving and facilitating the circulation in the internal organs. On the contrary, when the muscular system is inactive, the circulation is impeded in the viscera, which become congested and their veins dilated. This is frequently the case with the system of the portal vein; hence the passive congestions in the abdominal organs, especially in the sexual organs of women.

Again, the development of the muscular system increases also the energy of the heart's action, thus faciliting the circulation generally; whereas want of muscular exercise weakens the heart and retards the circulation.

It is, therefore, obvious that those methods are irrational, which, in the treatment of chronic diseases of women require prolonged rest and muscular inactivity, and that much may be gained by any mode of treatment that admits of muscular exercise."

:o:

### The Application of Earth in Surgery.

It was my good fortune during the winter to be residing in the same hotels, both at Nice and Rome, with the eminent American surgeon, Dr. Hewson, of Philadelphia, who brought to my notice the advantages attending the use of earth as a dressing in surgical cases. He told me he attributed his great success at the Pennsyl-

*Physioligie, 2 Auflage, 1872, p. 634.

